# Evolution of clonal populations approaching a fitness peak

**DOI:** 10.1098/rsbl.2012.0239

**Published:** 2013-02-23

**Authors:** Isabel Gordo, Paulo R. A. Campos

**Affiliations:** 1 Instituto Gulbenkian de Ciência, Oeiras, Portugal; 2 Departamento de Física, Universidade Federal de Pernambuco, Cidade Universitaria, 50670-901, Recife PE, Brazil

**Keywords:** experimental evolution, clonal interference, molecular clock, epistasis, fitness peak

## Abstract

Populations facing novel environments are expected to evolve through the accumulation of adaptive substitutions. The dynamics of adaptation depend on the fitness landscape and possibly on the genetic background on which new mutations arise. Here, we model the dynamics of adaptive evolution at the phenotypic and genotypic levels, focusing on a Fisherian landscape characterized by a single peak. We find that Fisher's geometrical model of adaptation, extended to allow for small random environmental variations, is able to explain several features made recently in experimentally evolved populations. Consistent with data on populations evolving under controlled conditions, the model predicts that mean population fitness increases rapidly when populations face novel environments and then achieves a dynamic plateau, the rate of molecular evolution is remarkably constant over long periods of evolution, mutators are expected to invade and patterns of epistasis vary along the adaptive walk. Negative epistasis is expected in the initial steps of adaptation but not at later steps, a prediction that remains to be tested. Furthermore, populations are expected to exhibit high levels of phenotypic diversity at all times during their evolution. This implies that populations are possibly able to adapt rapidly to novel abiotic environments.

## 1. Introduction

In asexual populations, the process of adaptation can be complex owing to interference between selected mutations. This is called the Hill–Robertson effect, which leads to increased rates of fixation of deleterious mutations (Muller's ratchet) and decreased rates of fixation of beneficial mutations (clonal interference, CI; [1]). Theoretical models predict that both the rate of fitness increase and the rate of molecular evolution will be constant (reviewed in Sniegowski & Gerrish [2]). These models are valid when the rate of environmental change is large and the supply of beneficial mutations virtually infinite. However, when the environment changes slowly, the rate of adaptation declines as the population adapts. Experimental evolution has provided indications that this can be the case. Indeed, the rate of increase of the competitive ability of populations of *Escherichia coli* declines considerably after a few thousands of generations [3]. Recently, a genomic analysis of the mutations associated with those changes suggested an interesting and paradoxical pattern of variation: while the rate of fitness change is highly nonlinear, the rate of molecular evolution is approximately constant over the period studied (20 000 generations) [3]. Because most mutations fixed have been shown to be advantageous, it is rather surprising that their rate of accumulation is constant.

Two other interesting patterns are observed in these and other experimentally evolved populations: the emergence of mutator alleles [4,5] and the occurrence of negative epistasis between beneficial mutations [6,7]. Mutators of intermediate strength emerge when the rate of fitness increase is already very small [4].

Here, we investigate whether these observed patterns can be expected under theoretical models of adaptation. A geometrical model of adaptation towards a single fitness peak was first developed by Fisher's geometrical model (FGM). It has been extended to study more general fitness functions, epistasis and drift load [8–10] and is successful in interpreting experimental data [10–14]. We consider FGM with intense CI and, using stochastic simulations, we study under what conditions it can predict those patterns. We consider different values of the parameter space, but focus on genomic mutation rates that are biologically realistic for bacteria [15].

## 2. Material and methods

In FGM, an organism is a point in an *n*-dimensional Euclidean phenotypic space, and a Gaussian fitness landscape is assumed. In simulations, each individual is represented by its phenotypic trait values (vector ***x*** = (*x*_1_,*x*_2_, … ,*x_n_*)), which yields fitness *W* = exp(−*Σ_i_x_i_*^2^), and by its number of mutations. Initially, all individuals have the same trait values, no mutations and fitness *W*_0_. Mutations are Poisson distributed with rate *U* and their effects modelled as random vectors, from a normal distribution with mean 0 and variance *σ*^2^. At the origin, all mutations are deleterious (each of effect *s*_d_) with mean *E*(*s*_d_) = −*nσ*^2^. Individuals contribute to the next generation according to their fitness.

We modify FGM by making the ad hoc assumption of a non-static optimum. Assuming that the organism is part of the environment and that when it changes, the environment changes [16], we extended FGM in the following way. During adaptation, many mutations arise per generation (in a typical microbial experiment [3], the number can easily reach 10^4^). This means that a small fraction of the population is composed of different genotypes at any given time. These mutations are mostly deleterious but occasionally beneficial. If organisms are adapting to the environment, then random fluctuations of the environment always occur. We model this by assuming that the phenotypic optimum in FGM changes stochastically, i.e. performs a random walk with no preferential direction. We call this model the shaking optimum model. To simulate it, we assume that the optimum at time *t* = 0 is set at position ***x***_opt_(0) = (0, … ,0), and at subsequent generations ***x***_opt_(*t*) = ***x***_opt_(*t* − 1) − *D**x***, with *D**x*** **=** (*Dx*_1_,*Dx*_2_, … ,*Dx_n_*) the displacement vector, where *Dx_i_* is a random deviate from a normal distribution with mean zero and variance *v* (ranging within 10^−7^ to 10^−5^). At least 10 simulations are run for each set of parameters.

To study the probability of fixation of mutators during the adaptive walk, we introduce into the population a single clone (modifier; [17]) that has a higher mutation rate (*s*_mut_*U). The mutator is introduced after the population has evolved for *T* generations and its fate is followed during this adaptive walk. Many independent introductions are performed to estimate the probability of fixation for each time *T*. The mutator is considered to have undergone hitchhiking if its probability of fixation is above 1/*N* (that of a neutral allele).

## 3. Results

### (a) Dynamics of adaptation under Fisher's geometrical model with clonal interference

**Figure 1.**
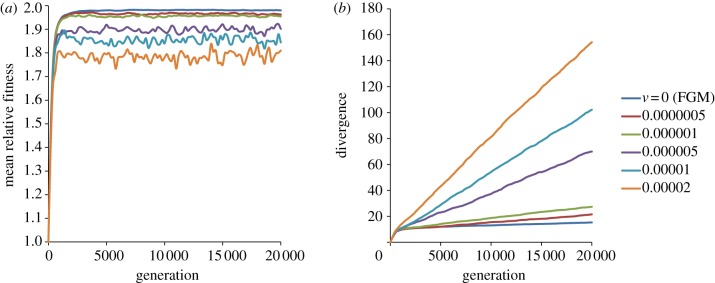
(*a*) Dynamics of fitness increase and (*b*) divergence (number of mutations accumulated) under FGM with a shaking peak. *W*_0_ = 0.5, *N* = 10^7^, *U* = 0.001, *σ*^2^ = 0.001 and *n* = 20. The optimum shakes every generation with a variance *v*. In the limit *v* → 0, we recover the classical FGM, where the mean effect of mutations at the optimum is −2%.

We study the evolution of clonal populations where the mutation input is large (NU≫1) and both beneficial and deleterious mutations occur, so we study FGM under intense CI. FGM with a static optimum generally predicts that the mean population fitness increase is rapid and then achieves a plateau. The rate of molecular evolution parallels the fitness increase, and the accumulation of mutations stops after the fitness plateau is achieved (figure 1). Simulations with different sets of parameters predict that organisms with bigger phenotypic complexity (larger *n*) adapt more slowly and that in small populations mean fitness increases at a slower pace [8–10], as expected (see the electronic supplementary material). In FGM, the mean effect of deleterious mutations changes along the walk towards the peak. In the electronic supplementary material, supporting information, we present simulations with different selection coefficients. As expected, a nearly constant rate of mutation accumulation is observed only in cases in which the majority of mutations are effectively neutral. However, this is also accompanied by a very slow and nearly constant rate of fitness increase. From other simulations, similar qualitative behaviours were observed leading to the conclusion that FGM, in its classical form, generally does not predict an abrupt fitness increase accompanied by a constant rate of molecular change.

We now consider the shaking optimum. If organisms are adapting to the environment that includes themselves [16], then random fluctuations (of variance *v*) of the optimum are always occurring. Figure 1 shows that when the fluctuations are very small (low values of *v*), we observe the same patterns as in the classical FGM: an initial high rate of both fitness increase and divergence at the molecular level, followed by a fitness plateau and a drastic reduction of the rate of molecular evolution. Strikingly, as the optimum diffuses around its initial location, we observe similar fitness dynamics but quite a different pattern at the molecular level. Although at the fitness level populations reach a plateau, at the molecular level, divergence increases at a constant rate. Importantly, the fitness plateau is dynamic, characterized by oscillations around a mean. Figure 1 and the simulations with other parameters (see the electronic supplementary material) all illustrate three important features: (i) an initial rapid increase of fitness, which means that populations become well adapted to the environment in the earlier stages; (ii) the plateau is only a time average—in fact, mean fitness fluctuates through time (interestingly, strong fluctuations in fitness are also observed in long-term experimentally evolved lines; [3]); and (iii) a constant rate of molecular evolution underlying the fitness trajectory. These features are similar to a Red Queen scenario for a single species [18]; at the molecular level, evolution is not stopping, whereas the fitness level remains unaltered.

### (b) Invasion of mutator alleles, epistasis and trait variation

**Figure 2.**
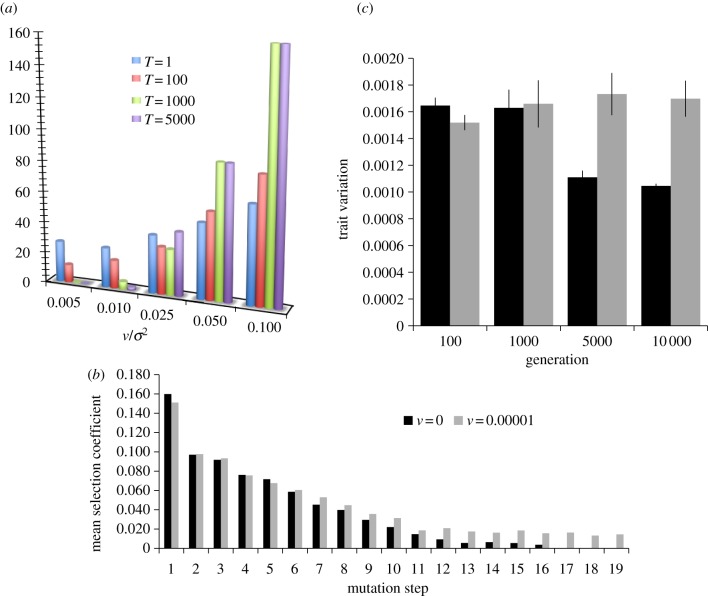
(*a*) Relative probability of fixation of a mutator (mutator advantage), with strength *s_mut_* = 100, introduced at generation *T* (different bar colours). *N* = 10^4^ *σ*^2^ = 0.001, *n* = 20, *U* = 0.001, *W*_0_ = 0.8. (*b*) Mean fitness effect of fixed mutations along the adaptive walk. The black bars represent a static landscape (*v* = 0), and the grey bars a randomly shaking optimum. *N* = 10^6^ *σ*^2^ = 0.001, *n* = 20, *U* = 0.001, *W*_0_ = 0.5. (*c*) The mean within-population variance in trait values observed along the adaptive walk (at generation 10 000 the fitness plateau was already achieved). Black bars represent a static landscape (*v* = 0), and grey bars a randomly shaking optimum (*v* = 10^−5^). *N* = 10^5^ *σ*^2^ = 0.001, *n* = 20, *U* = 0.001 and *W*_0_ = 0.5.

We studied the advantage of mutator alleles along the adaptive walk. Figure 2*a* shows the relative probability of fixation of a mutator during the adaptive walk. In static fitness landscapes (*v* → 0), the probability of fixation of mutators decreases as the population adapts. However, for a shaking optimum, the opposite can occur. There, the probability of fixation of the mutator is lower at the beginning of the adaptive walk and then becomes constant as populations wander around the fitness plateau. The probability of fixation also increases with the magnitude, *v*, of the shaking peak oscillations. Consistent with the hypothesis that mutators hitchhike with beneficial alleles, we find that the advantage of the mutator is highest in larger populations (see the electronic supplementary material).

The pattern of epistasis between beneficial alleles can be studied by measuring the mean effect of the mutations that get fixed at each step of climbing the landscape in FGM. Figure 2*b* shows that, during the first step of the adaptive walk, very strong effect mutations fix. Afterwards, the mean effect of fixed mutations becomes smaller. However, in latter stages of adaptation, the static and the shaking optimum models are distinct: whereas in the static, negative epistasis is the rule (see the electronic supplementary material) in the shaking, no epistasis is detected. In other words, after an initial rapid fitness increase, which is characterized by negative epistasis in both models, multiplicative fitness is expected under the shaking optimum, where beneficial mutations are still available. This prediction can be tested in experimental lines by studying those mutations that have fixed later in the adaptive process. In addition, the levels of variation at the trait level are also distinct. Figure 2*c* shows that (under the shaking peak) the phenotypic diversity is kept large along the adaptive walk, particularly after a dynamic fitness plateau has been achieved (grey bar at generation 10 000). This contrasts with the expectation under the static FGM, where phenotypic diversity is expected to decrease as the populations adapt. Therefore, in the landscape with a shaking peak, populations exhibit high levels of phenotypic diversity, implying that they can more readily respond to novel selection pressures from changes in the abiotic environment.

## 4. Discussion

We studied FGM under CI and extended it to include the possibility of a non-static optimum. Such extension predicts the seemingly paradoxical pattern of phenotypic and molecular change observed in experiments. It predicts a dynamic fitness plateau and a high probability of invasion of mutator alleles in the initial stages (as observed), but also when populations have reached such a plateau (also observed). It also implies that some amount of non-transitive fitness interactions may be detectable when competing different clones with the ancestral population, as well as a change in the pattern of epistasis along the adaptive walk. While some evidence for non-transitive fitness interactions has been observed in experimentally evolved populations [19], the pattern of epistasis between mutations along the walk has not yet been measured.

In a recent genotypic model of dynamic fitness landscapes (seascapes), conditions were found where a rapid rate of fitness increase could be accompanied by a much slower rate of change at the molecular level [20]. It would be interesting to explore if that and other models [21] can also capture the main observations made in experimental evolution with asexual microbes.
